# Ultra-Reliable Communication for Critical Machine Type Communication via CRAN-Enabled Multi-Connectivity Diversity Schemes †

**DOI:** 10.3390/s21238064

**Published:** 2021-12-02

**Authors:** Binod Kharel, Onel Luis Alcaraz López, Hirley Alves, Matti Latva-aho

**Affiliations:** Centre for Wireless Communications, Faculty of Information Technology and Electrical Engineering, University of Oulu, 90014 Oulu, Finland; Onel.AlcarazLopez@oulu.fi (O.L.A.L.); Hirley.Alves@oulu.fi (H.A.); matti.latva-aho@oulu.fi (M.L.-a.)

**Keywords:** CRAN, diversity, MTC, multi-connectivity, reliability, ultra-reliable communication, URLLC

## Abstract

This paper focuses on edge-enabled cloud radio access network architecture to achieve ultra-reliable communication, a crucial enabler for supporting mission-critical machine-type communication networks. We propose coordinated multi-point transmission schemes taking advantage of diversity mechanisms in interference-limited downlink cellular networks. The network scenario comprises spatially distributed multiple remote radio heads (RRHs) that may cooperate through silencing, or by using more elaborated diversity strategies such as maximum ratio transmission or transmit antenna selection to serve user equipment in the ultra-reliable operation regime. We derive an exact closed-form expression for the outage probabilities and expected values of signal-to-interference ratio for silencing, transmit antenna selection and maximum ratio transmission schemes. We formulate rate control and energy efficiency under reliability constraints to test the performance and resource usage of the proposed schemes. Furthermore, we study the impact on average system sum throughput with throughput-reliability trade-off under cooperative communication. Extensive numerical analysis shows the feasibility of ultra-reliable communication by implementing diversity schemes with RRHs cooperation.

## 1. Introduction

Fifth-generation (5G) and beyond networks will have multi-service communication. These multi-services are not only about higher data rate applications such as enhanced mobile broadband (eMBB) but are also designed to serve machine type communication (MTC) [1]. MTC addresses two main scenarios: massive MTC (mMTC) and ultra-reliable low-latency communication (URLLC) [2]. Massive MTC deals with the massive number of MTC devices in a given area, while URLLC is a novel service class aiming to address stringent latency and reliability requirements [3]. There are several potential URLLC applications, such as factory automation [4], process automation [4], intelligent transport systems [4], automated guided vehicles (AGV) [5] and smart grids [6] which require high reliability (e.g., frame error rates of $10^{-5}$ to $10^{-9}$, respectively depending upon the application) while guaranteeing very low latency [6,7].

URLLC can be studied from two aspects: ultra-reliable communication (URC) and low latency communication (LLC). The author in [8] introduces URC over the long term, in which the required latency is >10 ms, and URC in the short term, referring to problems with very stringent latency requirements, below 10 ms. In this work, we focus on URC over the short term that delivers a message from source to destination with guaranteed high-reliability [9]. Some challenges related to achieving ultra-reliability are link degradation, fading channel, shadowing, and interference which increases the probability of outage in wireless communication communication [10,11]. From the ultra-reliable communication perspective, all these challenges add further complexity to the physical layer design [12]. Hence, in the next-generation wireless services, ensuring ultra-reliability for mission-critical applications is a challenging task.

One of the identified solutions to overcome this challenge and enhance system reliability is multi-connectivity (MC). The basic idea of MC is to serve or send the packets to the user via multiple links through the spatial or frequency domain. Multi-connectivity adopts spatial diversity with the concept of cloud radio access network (CRAN) to ensure ultra-reliability through cooperative solutions [13]. In the CRAN, a large amount of signal processing and computing is performed in a distributed manner; base band units (BBUs) act as virtual base stations (BSs) responsible for resource allocation and centralized baseband processing, while remote radio heads (RRH) change baseband signal to radio frequency signal for ahead transmission to the users [14,15]. CRAN has high-speed optical fiber links in the front haul that can support low latency and high-capacity communication, both of which lead to improved network performance [16].

Motivated by the above concerns, the aim of this paper is to achieve ultra-reliable region operation through interference management by the cooperation/coordination of RRH for downlink transmission through CRAN using multiple spatially distributed RRH connections. This kind of framework with edge-enabled multi-coordinated transmission schemes is beneficial, for example, in an industrial environment where various types of devices are present such as robots, AGV, sensors, and actuator devices which rely on URC [17]. We exploit MC through the maximum ratio transmission (MRT) and transmit antenna selection (TAS) scheme, or by reducing interference through silencing in an interference-limited downlink cellular network with RRHs’ cooperation, and analyze the performance of different diversity schemes to ensure URC for critical MTC. Machine learning techniques are also identified as a potential candidate for radio resource management for wireless networks for some critical applications [18,19]. However, due to the novelty of the critical MTC network, the main limitation of applying machine learning tools in these scenarios is the lack of an appropriate and adequate dataset for their precise application. Indeed, we intend to cover the analysis with machine learning solutions as part of future works. Hence, we choose the above transmission schemes and focus with these diversity-based techniques on how much performance gain is achieved under diverse system parameters to support critical MTC.

### 1.1. Relevant Works

Many recent studies have focused on the reliability and latency aspects of URLLC as essential requirements in applications such as the industrial internet of things (IIoT), virtual reality, and autonomous vehicles [20]. Popvoski et al. [7,21] discuss the communication theoretic principles as a critical component in supporting URLLC system design, such as the use of various diversity resources, the design of packets, and access protocols. In this context, potential enablers for high reliability include diversity, multi-connectivity, and modulation and coding schemes. In this work, we focus on MC-enabled diversity schemes. Diversity is the capability to exploit channel variations over time, frequency, and space for communication robustness, and play an essential role in boosting reliability. We can achieve diversity gains using time, frequency, and spatial diversity mechanisms. Time diversity considers re-transmission, e.g., hybrid automatic repeat requests (HARQ). Time diversity is usually hard to implement because of the latency constraint presented in mission-critical applications [22]. Frequency diversity uses transfer over sub-band or multiple carrier frequencies separated by coherence bandwidth for one transmission. However, due to bandwidth constraints, and to achieve high reliability, using frequency diversity requires impractically high signal-to-noise ratios in practical channel condition [23]. Hence, spatial diversity seems to be more attractive and considered as one of the key enablers to improve network performance as a low cost, and power-efficient solution [24].

In spatial diversity, multiple antennas physically separated from each other are used to transmit to the user. The authors in [25] discuss the concept of multi-connectivity to achieve high reliability and increase the performance of wireless networks using standard diversity schemes like joint decoding, selection combining, and maximum ratio combining. The work in [26] proposed interface diversity, where each interface is based on different technologies to offer URLLC without intervention in baseband/physical layer design. Furthermore, cooperative diversity emerges as a workable alternative to direct communication [27,28]. Dual connectivity achieves superior reliability at the cost of lower mean spectral efficiency (because of redundancy) as potential solutions concerned with high-reliability [29,30]. Reference [31] propose a multi-connectivity concept in CRAN to reduce mobility-related link failures and cell-edge degradation. However, small cell deployment is necessary for dual and multi-connectivity, which would lead to an increase in interference over the network, and the necessary deployment problem becomes complicated. To overcome this problem in our work, we use interfering nodes to cooperate or coordinate to transmit to the intended user equipment (UE). The cooperation techniques turn interference from foe to friend while having a diversity gain with the CRAN architecture.

Furthermore, ref. [32] discuss CRAN technology to support URLLC, lowering the latency of URLLC traffic with the functional split of the central unit and the radio unit. This decentralization is an alternative where distributed RRHs can improve cloud accessibility with low latency and high-reliability [33]. The rate allocation problem in downlink cellular networks with stringent reliability constraints is investigated in [34]. This work concludes that an ultra-reliable region is achieved by using multiple antennas at the receiver for finite and infinite blocklength coding. Motivated by the merits of CRAN [31,32,33], we establish an MC framework through spatially distributed RRHs, using cooperation strategies enabled by CRAN to achieve URC in an interference-limited communication scenario. In a nutshell, the main distinction from [23,25,26,34] works is that we analyze typical link performance under Rayleigh fading, where multiple transmitting RRHs operating in the neighbourhood interfere with the typical link cooperate via CRAN, reducing the transmission interference for the desired UE and improving system reliability.

### 1.2. Our Contributions

The previous generation of networks with distributed antenna systems exploited spatial diversity to improve spectral efficiency, continuing in the current generation of networks. However, in this paper, we provide novel contributions in the state of the art of 5G beyond communication, and extensively investigate the performance of proposed spatial diversity transmission techniques considering interference and cooperation strategies to achieve URC for critical machine type communication networks. We further extend [35] introducing one more transmission scheme TAS with silencing and attain-close form expression of outage probability. Furthermore, we analyze the expected value of the signal-to-interference ratio (SIR), energy efficiency (EE), and throughput-reliability trade-off. We evaluate the performance and resource usage among the considered transmission schemes. We remark that ensuring high reliability using multiple node redundant transmission is also included in the study of enhancements for URLLC support in the 5G Core network in 3GPP (Release 16) [36]. The major contributions are summarized as follows:

- We analyze UE’s performance when operating under full interference, silencing, TAS with silencing, and MRT in terms of the outage probability. We attain the accurate closed-form expressions of the outage probability for the distribution of the SIR of each scheme.
- We calculate the expected value of SIR and attain closed form solutions for each scheme. We show that MRT has higher SIR compared with the other schemes, whereas silencing and TAS with silencing show similar SIR with the same number of cooperating RRHs.
- We address the rate control problem constrained by target reliability constraints for the proposed schemes. We show that for all considered schemes, transmission rate increases with the increase in cooperating RRHs, while MRT offers a higher transmission rate among them for the same target reliability and for the same number of cooperating RRHs.
- We test energy efficiency (EE) for each scheme. We show that EE for all considered schemes increases with an increase in the cooperating RRHs, while MRT schemes have higher EE among considered schemes for the same target reliability and with the same number of cooperating RRHs.
- We analyze the minimum number of cooperating RRHs required to achieve a certain reliability level for all these considered schemes. We show that for the same number of cooperating RRHs, the MRT scheme achieves higher reliability levels. In contrast, this level is unattainable with other schemes.
- Finally, we analyze the trade-off between average system throughput and reliability to test CRAN network-level performance with considered transmission schemes through Monte Carlo simulations. We show that MRT attains a higher reliability level for the same number of cooperating RRHs with reduced throughput than the silencing and TAS.

The rest of the paper is organized as follows. Section 2 explains the system model and highlights the main assumption along with transmission schemes. Section 3 presents the definition of diversity and reliability, outage formulation, rate control, energy efficiency, and throughput-reliability trade-off analysis under reliability constraints. Section 4 shows numerical analysis. Finally, Section 5 concludes the paper.

Notation: Uppercase and lowercase letters denote random variables (RVs) and their realizations, unless stated otherwise. The probability density function (PDF) and cumulative distribution function (CDF) of RV *X* is denoted by $\;f_{X}\left(x\right)$ and $F_{X}\left(x\right)$, respectively. E[.] denotes the expectation operation. X∼Exp(1) is an exponential RV with the $F_{X}\left(x\right)\;=\;1-e^{-x}$, while $\;Y\;∼\Gamma (n,\frac{1}{n})$ with $\;f_{Y}\left(y\right)\;=\frac{y^{n-1}\exp^{-y}}{(n-1)!}$. Additionally, Γ(p,x)=$\int_{x}^{\infty }t^{p-1}e^{-t}dt$ is the upper incomplete gamma function, and ${}_{2}F_{1}\left(a,b;c;d\right)$ denotes the Gaussian regularized hypergeometric function [37].

## 2. System Model

### 2.1. Network Model and Operation

**Figure 1:**
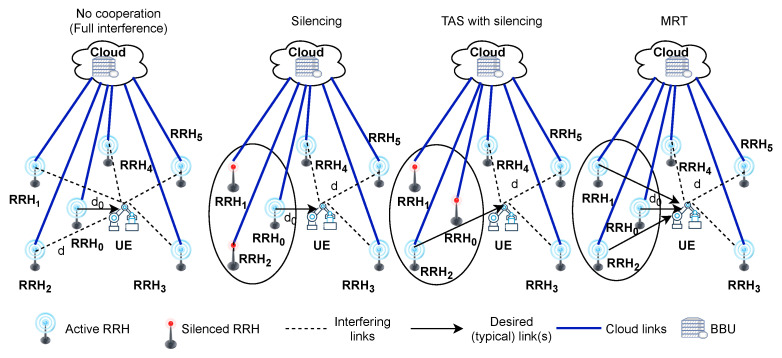
Illustration of the CRAN system model for different transmission schemes with a typical link and N = 5 interfering RRHs.

We consider a CRAN system as illustrated in Figure 1, where all the RRHs are connected to an edge cloud consisting of a baseband unit (BBU) pool via cloud links with high-bandwidth and low latency communication. In the setup, N+1 RRHs, i.e., RRH${}_{0}$, RRH${}_{1}$, ⋯, RRH${}_{N}$ are spatially distributed in a given area $\;A\;\subseteq R^{2}$. We assume that all RRHs are using the same spectrum resources (i.e., time and frequency) when transmitting to their corresponding UEs. We consider the link between RRH${}_{0}$ and UE as a typicallink. Meanwhile, we assume that link from RRH${}_{j}$ to UE for all j∈1,⋯,N as (potentially) interfering links to the typical link. We use the notation $d_{0}$ to denote the distance between the RRH${}_{0}$ and the UE, while *d* denotes the distance from all other RRH${}_{j}$ to the UE. The UE of interest is served by the closest RRH, while other RRHs are uniformly distributed on the perimeter of a circle (with radius $d>d_{0}$) centered on the UE (Notice that in real-world setups, at any instant of time, the UE could be at any random location. For analytical tractability, we have eased this constraint by assuming equal distances to interfering nodes such that all the interference from other interfering RRHs are assumed from the same nodes). We focus on the typical link performance while all the other RRH${}_{j},\;j\;=\;1,\cdots ,N$ are using the same channel to transmit data to their corresponding UEs. We carry the analysis for the typical link performance when the remaining RRHs are:

- not cooperating with the typical link through CRAN, i.e., no cooperation (full interference scenario as shown in Figure 1);
- cooperating with the typical link through the CRAN to serve the UE (BBU at CRAN enables coordinated multi-point transmission and cooperative solutions, similar to scenarios described in [12,14,25,29]). Under the cooperation, we proposed silencing, TAS with silencing and the MRT scheme as shown in Figure 1 which is explained in detail in the following Section 2.2.

### 2.2. Transmission Schemes

We studied the following three strategies under cooperation.

- Silencing: CRAN silences some of the interfering RRHs, thus mitigating interference to enhance the system performance of the typical link. At a silenced RRH, the transmission signals are completely turned off, which helps to boost the SIR in the victim cell, and it has been proposed for 5G [38].
- TAS with silencing: CRAN selects the best channel for transmission among the typical link and cooperating RRHs. After selecting the best channel, it forces all the cooperating RRHs to remain silent. This scheme presents some diversity gain and has optimal reception reliability at the UE.
- MRT: CRAN jointly transmits from the typical link and cooperating RRHs to serve the UE. This scheme provides high optimal reception reliability and significant diversity gain to cope with very stringent reliability constraints and fading channel impairments.

We assume that during transmission, a proper amount of pilot signal accompanies all transmission schemes [23]. Hence, TAS and MRT require 1-bit feedback to select among the best link or jointly transmit to the desired UE. However, in silencing, CRAN sends a decision to the cooperating RRH to remain silent during the beginning of every downlink transmission slot, which avoids the use of precise control and extra signalling. Thus, proposed schemes do not incur heavy signalling overhead burden and hence are feasible for upcoming 5G systems [39,40]. MRT is considered as a reference scheme to provide optimal performance with higher transmit diversity gain compared to TAS and silencing [41]. However, MRT can be costly compared to TAS and silencing at the implementation side, but this scheme is being implemented in practice [25]. Meanwhile, in the case of a limited feedback system avoiding the complexity compared to MRT, TAS provides significant performance gains [42,43].

### 2.3. Communication Model

We assume a multi-node downlink communication scenario where both the RRHs and UE are equipped with a single antenna. The channel is modeled by distance-dependent path loss and small-scale fading. We assume the channel undergoes quasi-static Rayleigh fading and the path loss exponent is α. Because of latency constraints, all processing is done centrally at the CRAN. Similar to [40], we assume that full channel state information (CSI) is available at CRAN BBU. We denote the i.i.d squared-envelope fading coefficients of the typical, cooperating and interfering link as $h_{0}$,$h_{c}$,$h_{j}∼\mathrm{Exp}\left(1\right)$, respectively. We consider that each RRH transmits with fixed unit power, and there is a dense network deployment such that the system is interference limited. Therefore, the noise is negligible compared to the sum interference [44].

Under these assumptions and settings, the Signal-to-Interference Ratio (SIR) at UE for different transmission schemes as outlined in Section 2.2 is given by,

(1) $$\begin{array}{c} \mathrm{SIR}=\left\{\begin{array}{cc} \frac{h_{0}d_{0}^{-\alpha }}{\;I_{j}}, & \mathrm{for}\;\mathrm{silencing}\\ \frac{\max(h_{0}d_{0}^{-\alpha },h_{c}d^{-\alpha }),c=1,\cdots ,k}{\;I_{j}}, & \mathrm{for}\;\mathrm{TAS}\\ \frac{h_{0}d_{0}^{-\alpha }+\sum_{c=1}^{k}h_{c}d^{-\alpha }}{\;I_{j}}, & \mathrm{for}\;\mathrm{MRT} \end{array}\right. \end{array}$$

Note that while writing SIR expression in (1) it is assumed that first *k* RRHs are cooperating with the transmission of the typical link, the rest of the RRHs are interferers, and $I_{j}=\sum_{j=k+1}^{N}h_{j}d^{-\alpha }$ is the sum of interference from the other interferers. We assume that, at a given time, there are other active users being served by non-cooperating RRHs using the same spectrum resources. Hence, the total sum interference $I_{j}$ takes into account the collective interference from all transmitting streams corrupting the typical link.

## 3. Diversity and Reliability

Diversity is the capability to exploit channel variations over time, frequency, and space for communication robustness. We study spatial diversity following the aforementioned transmission schemes to achieve URC. Reliability is the successful transmission of a certain amount of data from a source to a destination within a latency budget. Our working definition of reliability is the focus with 3 GPP definition [45] as “capability of transmitting an amount of traffic within a predetermined time duration with high success probability. The minimum requirement for reliability is $1-10^{-5}$ success probability of transmitting over layer two protocol data unit of 32 bytes within 1 ms”. In this sequel, using the SIR expressions defined in (1), we calculate the outage probability for each of the considered schemes. Furthermore, to elaborate more on the system performance and resource usage under the reliability constraint, we make further analysis on the expected value of SIR, rate control, energy efficiency and throughput-reliability with the considered system model.

### 3.1. Outage Probability Analysis

In order to calculate the outage probability, we proceed to calculate the CDF of the SIR as $P\left(\mathrm{SIR}<\theta \right)=F_{\mathrm{SIR}}\left(\theta \right)$ and formulate it for the different transmission schemes in consideration. Note that the threshold is $\theta =2^{r}-1$ [34], where *r* is the target rate. Let us define $\delta =\frac{d_{0}}{d}$, the ratio between the typical link distance to the other interfering RRH distances from UE.

We analyze the system performance in terms of outage probability (i.e., 1−Reliability) for all considered transmission schemes. In the following subsection, we provide closed-form solutions for the outage probabilities and expected values. We also define performance metrics such as rate control, energy efficiency, and throughput-reliability trade-off for the considered transmission schemes to test performance and resource usages. In the sequel, the analytical results are then corroborated via Monte Carlo simulations and discussed in Section 4.

#### 3.1.1. Silencing

In this scheme, CRAN silences *k* interfering RRHs out of *N*, and RRH${}_{0}$ is serving the UE. This sort of silencing scheme reduces the total interference experienced at the UE side. With this strategy, we can rewrite the SIR for the silencing scheme from (1) as

(2) $$\begin{array}{c} {\mathrm{SIR}}_{S}=\frac{h_{0}d_{0}^{-\alpha }}{\sum_{j=k+1}^{N}h_{j}d^{-\alpha }}, \end{array}$$

and elaborate the outage probability in Theorem 1.

**Theorem** **1.** *The CDF of the SIR when the CRAN employs the silencing scheme to serve the UE is*

(3) $$\begin{array}{c} F_{{\mathrm{SIR}}_{S}}\left(\theta \right)=1-{\left(1+\delta^{\alpha }\theta \right)}^{k-N}. \end{array}$$

**Proof.**  Please refer to Appendix A. □

The expected value of ${\mathrm{SIR}}_{S}$ is given as

(4) $$\begin{array}{cc} E[{\mathrm{SIR}}_{S}] & \overset{}{=}\int_{0}^{\infty }\left(1-F_{{\mathrm{SIR}}_{S}}\left(\theta \right)\right)d\theta ,\\ \; & \overset{\left(a\right)}{=}\int_{0}^{\infty }{\left(1+\delta^{\alpha }\theta \right)}^{k-N}d\theta ,\\ \; & =\frac{\delta^{-\alpha }}{N-(k+1)}. \end{array}$$

where (*a*) comes after substituting the respective CDF. Solving the integral in (*a*) we get (4). In (4) as the value of *k* increases, the factor (N−k−1) is reduced, thereby increasing $E[{\mathrm{SIR}}_{S}]$.

#### 3.1.2. Transmit Antenna Selection with Silencing

In this scheme, the CRAN chooses the best channel among the RRH${}_{0}$ and the *k* cooperating RRHs in order to serve the UE. After selecting the best channel, the RRHs that are cooperating remain silent while the non-cooperating RRHs cause interference. This scheme reduces the interference and improves overall average system performance by enabling diversity transmission. We can evaluate the SIR in case of TAS with silencing from (1) as

(5) $${\mathrm{SIR}}_{\mathrm{TAS}}=\frac{\max(h_{0}d_{0}^{-\alpha },\left(h_{1},\cdots ,h_{k}\right)d^{-\alpha })}{\sum_{j=k+1}^{N}h_{j}d^{-\alpha }},$$

while expressing the outage probability in Theorem 2,

**Theorem** **2.** *The CDF of the SIR in the case when UE is served through TAS with silencing is*

(6) FSIRTAS(θ)=∑t=0kkt(−1)t(1+θt)k−N−1+θt+θδαk−N.

**Proof.**  Please refer to Appendix B. □

The expected value of ${\mathrm{SIR}}_{\mathrm{TAS}}$ is given as

(7) E[SIRTAS]=∫0∞(1−FSIRTAS(θ))dθ,=(a)∫0∞1−∑t=0kkt(−1)t(1+θt)k−N−1+θt+θδαk−Ndθ,=δ−αN−(k+1)+∑t=1kkt(−1)tδαt(N−k−1)(δα+1).

where (*a*) comes after substituting the respective CDF. Solving the integral in (*a*) and with simplification we reached (7). The first term in (7) is the same as (4) whereas the second term gives some of the diversity gain from TAS with slight increments in $E[{\mathrm{SIR}}_{\mathrm{TAS}}]$ as compared to silencing.

#### 3.1.3. Maximum Ratio Transmission

MRT is the scheme where the typical RRH${}_{0}$, as well as the *k* cooperating RRHs, are jointly coordinated in transmission to the UE. Under this scheme, we define the SIR from (1) as

(8) $$\begin{array}{c} {\mathrm{SIR}}_{\mathrm{MRT}}=\frac{h_{0}d_{0}^{-\alpha }+\sum_{c=1}^{k}h_{c}d^{-\alpha }}{\sum_{j=k+1}^{N}h_{j}d^{-\alpha }}, \end{array}$$

and we express the outage probability in Theorem 3.

**Theorem** **3.** *The CDF of the SIR in the case when UE is served through MRT is*

(9) $$\begin{array}{cc} F_{{\mathrm{SIR}}_{\mathrm{MRT}}}\left(\theta \right) & =\frac{\Gamma (N)}{\Gamma (N-k)}\theta^{k}{\left(1+\delta^{\alpha }\theta \right)}^{-N}\times ({\left(1+\delta^{\alpha }\theta \right)}^{N}{}_{2}F_{1}\left(k,N;1+k;-\theta \right)\\ \; & -{}_{2}F_{1}\left(k,N;1+k;\frac{(-1+\delta^{\alpha })\theta }{1+\delta^{\alpha }\theta }\right)). \end{array}$$

*valid for all 1≤k≤N and δ<1.*

**Proof.**  Please refer to Appendix C. □

The expected value of ${\mathrm{SIR}}_{\mathrm{MRT}}$ is given as

(10) $$\begin{array}{cc} E[{\mathrm{SIR}}_{\mathrm{MRT}}] & \overset{}{=}\int_{0}^{\infty }(1-F_{{\mathrm{SIR}}_{\mathrm{MRT}}}(\theta ))d\theta ,\\ \; & \overset{\left(a\right)}{=}\int_{0}^{\infty }(1-(\frac{\Gamma (N)}{\Gamma (N-k)}\theta^{k}{\left(1+\delta^{\alpha }\theta \right)}^{-N}\times ({\left(1+\delta^{\alpha }\theta \right)}^{N}{}_{2}F_{1}\left(k,N;1+k;-\theta \right)\\ \; & -{}_{2}F_{1}\left(k,N;1+k;\frac{(-1+\delta^{\alpha })\theta }{1+\delta^{\alpha }\theta }\right))))d\theta ,\\ \; & =\frac{\Gamma \left(N-k-1\right)\left(k+\delta^{-\alpha }\right)}{\Gamma (N-k)} \end{array}$$

where (*a*) comes after substituting the respective CDF. Using the ®Mathematica integral package to solve the hypergeometric function in (*a*), we have (10). In (10), as the value of *k* increases, the numerator increases proportional to the *k* and this provides additional diversity gain in MRT with increases in $E[{\mathrm{SIR}}_{\mathrm{MRT}}]$ as compared to TAS and silencing.

**Remark** **1.** *Notice that by taking k=0 when there is no cooperation, all the considered transmission schemes represent the case of full interference.*

### 3.2. Rate Control under Reliability Constraints

In this section, we define rate control to estimate the target rate for the pre-defined reliability levels. The objective of the analysis is to find the optimal target rate while using the minimum number of cooperating RRHs, which is beneficial, more practical and reduces the complexity, and can ensure some level of system performance.

**Lemma** **1.** *The constant transmission rate for silencing scheme that guarantees an outage probability not above $\epsilon_{th}$ is*

(11) $$\begin{array}{c} r=\log_{2}\left(\frac{\epsilon_{th}^{-\frac{1}{N-k}}-1}{\delta^{\alpha }}+1\right). \end{array}$$

**Proof.**  Note that (11) is the final closed-form analytical solution for rate control analysis which comes directly after solving $F_{\mathrm{SIR}}S\left(\theta \right)=\epsilon_{th}$, for θ using (3), where $\theta =2^{r}-1$. □

However, in the case of TAS with silencing and MRT schemes, it is difficult to simplify and invert (6) and (9). In order to evaluate rate analysis for these two transmission schemes we proceed solving numerically as

(12) $$\begin{array}{c} \underset{r>0}{\arg\;\max}\;F_{{\mathrm{SIR}}_{\mathrm{TAS}}}\left(\theta \right)=\epsilon_{th}, \end{array}$$

(13) $$\begin{array}{c} \underset{r>0}{\arg\;\max}\;F_{{\mathrm{SIR}}_{\mathrm{MRT}}}\left(\theta \right)=\epsilon_{th}, \end{array}$$

We resort to vpasolve implemented in MATLAB to evaluate (12) and (13). In the analysis, we calculate the maximum target transmission rate to satisfy the given reliability constraint $\epsilon_{th}$ imposing in the Equations (12) and (13), respectively. The detailed numerical analysis is discussed in the following Section 4.

### 3.3. Energy Efficiency (EE) under Reliability Constraints

The objective of the analysis is to find the energy efficiency of each scheme and discuss more on performance and resource usage. The basic definition of EE is [46]

(14) $$\begin{array}{c} EE\;\left[\mathrm{bits}/\mathrm{joule}/\mathrm{Hz}\right]=\frac{Data\;rate\;[\mathrm{bits}/s/\mathrm{Hz}]}{Power\;consumption\;[\mathrm{Joule}/s]} \end{array}$$

where data rate for silencing, TAs with silencing and MRT can be evaluated from (11)–(13) respectively. We adopt the unified power consumption model as proposed in [47]. This model approximates the BSs power consumption as a piecewise linear function of the transmit power $P_{T}$:

(15) $$\begin{array}{c} {\hat{P}}_{i}=\left\{\begin{array}{c} \eta P_{T}+P_{i,active}^{\mathrm{RRH}};\;\mathrm{if}\;{\mathrm{RRH}}_{i,active}\\ P_{i,sleep}^{\mathrm{RRH}},\;\mathrm{if}\;{\mathrm{RRH}}_{i,sleep} \end{array}\right. \end{array}$$

where η is a constant reflecting the power amplifier efficiency, feeder loss and other loss factor due to power supply and cooling for ${\mathrm{RRH}}_{i}$, $P_{T}$ is the transmit power, $P_{i,active}^{\mathrm{RRH}}$ $P_{i,sleep}^{\mathrm{RRH}}$ are the power consumption at a RRH corresponding to active and sleep modes, respectively. Typically, $P_{i,active}^{\mathrm{RRH}}>P_{i,sleep}^{\mathrm{RRH}}$ so that it is beneficial to silent RRH, whenever possible for energy saving. Based on the above power consumption model in (15), we can write the total power consumption as

(16) $$\begin{array}{c} \hat{P}=\sum_{i=0}^{k}{\hat{P}}_{i} \end{array}$$

where, *k* is the number of cooperating RRH. We assume $P_{T}$, $P_{i,active}^{\mathrm{RRH}}$ and $P_{i,sleep}^{\mathrm{RRH}}$ are same for all the RRHs. Based on (15) and (16) we can write the power consumption model for each considered scheme as

(17) $$\begin{array}{c} \hat{P}=\left\{\begin{array}{c} \eta P_{T}+P_{active}^{\mathrm{RRH}}+kP_{sleep}^{\mathrm{RRH}}\;\mathrm{for}\;\mathrm{silencing}\;\mathrm{and}\;\mathrm{TAS}\\ \left(k+1\right)\left(\eta P_{T}+P_{active}^{\mathrm{RRH}}\right),\;\mathrm{for}\;\mathrm{MRT}. \end{array}\right. \end{array}$$

As we can see from (17), as *k* increases, there is an increase in power consumption. However, consumption seems more in the case of MRT compared to silencing and TAS with silencing.

### 3.4. Throughput-Reliability Trade off

The objective of the analysis is to test the cost of considering a reliability-oriented system model that has to bear on its average system throughput while increasing the number of RRHs cooperation to guarantee ultra-reliability. We implement a Monte Carlo-based computer level simulation to study the fundamental trade-off between reliability and average system sum throughput in CRAN cooperating mode for each scheme, and discuss in Section 4. Note that it is difficult to evaluate this analytically. In the simulation, we test the reliability with the following metrics:

(18) $$\begin{array}{cc} Rel_{\phi }^{ref} & =1-P\left({\mathrm{SIR}}_{\phi }<\theta \right)=1-F_{{\mathrm{SIR}}_{\phi }}\left(\theta \right). \end{array}$$

where ϕ∈{S,TAS,MRT} respectively, ${\mathrm{SIR}}_{\phi }$ is given in (1) and $F_{{\mathrm{SIR}}_{\phi }}$ is the corresponding outage probability and θ is the SIR threshold. Next, we evaluate the average system sum throughput with the following metrics:

(19) $$\begin{array}{c} {\mathrm{TP}}_{\phi }=R_{\phi }^{ref}+R_{\phi }^{A}, \end{array}$$

where $R_{\phi }^{ref}$ is the rate of intended URLLC user and $R_{\phi }^{A}$ is the corresponding average sum rate of non-cooperating RRHs active users. Then,

(20) $$\begin{array}{c} R_{\phi }^{ref}={\mathrm{Rel}}_{\phi }^{ref}\log_{2}\left(1+\theta \right), \end{array}$$

(21) $$\begin{array}{c} R_{\phi }^{A}=E\left[\sum_{i=k+1}^{N}\log_{2}\left(1+{\mathrm{SIR}}_{\phi }^{i}\right)\right], \end{array}$$

Finally, from (19)–(21) we have that,

(22) $$\begin{array}{c} {\mathrm{TP}}_{\phi }={\mathrm{Rel}}_{\phi }^{ref}\log_{2}\left(1+\theta \right)+E\left[\sum_{i=k+1}^{N}\log_{2}\left(1+{\mathrm{SIR}}_{\phi }^{i}\right)\right]. \end{array}$$

Note that as *k* increases, the reliability of intended user increases while overall average system sum throughput decreases.

## 4. Numerical Analysis

In this section, we show the numerical results to corroborate the analytical derivations. Furthermore, we evaluate the system performance in terms of reliability, e.g., 1-$\epsilon_{th}$ for the considered schemes. In the analysis, we set α=3.5, based on practical radio propagation measurement in an industrial setup which corresponds to a dense setup [48]; whereas $\delta =\frac{d_{0}}{d}$ while $d_{0}<d$ thus 0<δ≤1, we arbitrarily chose δ=0.5, unless stated otherwise. The topology comprises N=10 RRHs located away from the UE of interest. We used a Monte Carlo simulation of $10^{7}$ runs (Note that a larger number of samples reduces the variance at the tail of the distribution at the cost of longer simulation time. The latter could be avoided by resorting to more sophisticated Monte Carlo methods, which is out of the scope of this work). Notice that when there is no cooperation, k=0 all the schemes revert to the case of full interference.

**Figure 2:**
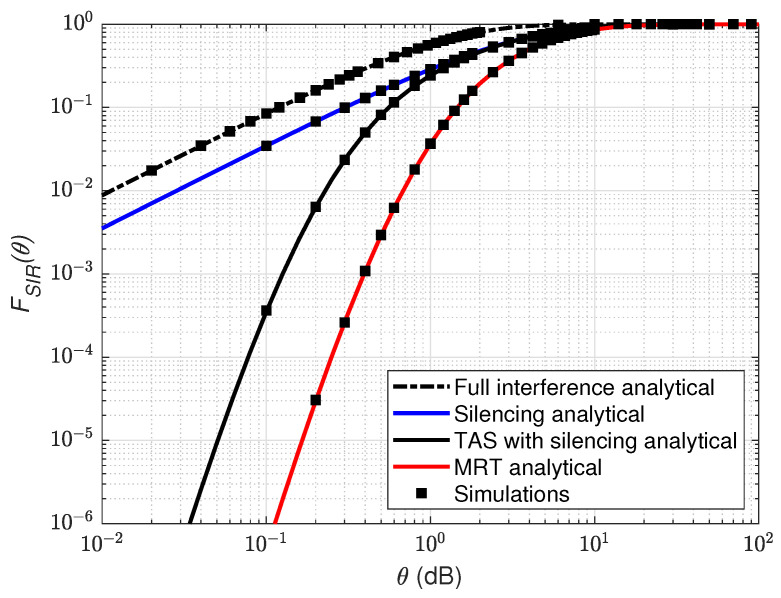
Overview of SIR distribution for full interference, silencing, TAS with silencing and MRT schemes as a function of θ. We set k=6 RRH in coordination.

Figure 2 illustrates the CDF of SIR distribution as a function of threshold θ (dB) for the considered transmission schemes with k=6 cooperating RRHs. As shown, for k=6 the left tail distribution of the MRT scheme is already exceeding the outage probability value of $10^{-5}$ as compared to the silencing scheme and TAS with silencing schemes. While with the k=6, RRHs silenced the left tail of the silencing scheme exceeds $10^{-2}$ in comparison with full interference. Similarly, the results seem to be better with the TAS with silencing scheme, where the left tail goes beyond $10^{-5}$ for a higher threshold of θ compared to the silencing and the full interference scenario. The MRT and TAS with silencing curves seemed to attain higher values of θ than the silencing and the full interference schemes, which shows significant performance gain and a higher diversity gain at the UE. However, MRT curves bend to a higher SIR threshold in comparison with the TAS scheme because joint transmission with cooperating RRHs in MRT enhances the SIR gain at the UE. Finally, we validate the analytical results via Monte Carlo simulations.

**Figure 3:**
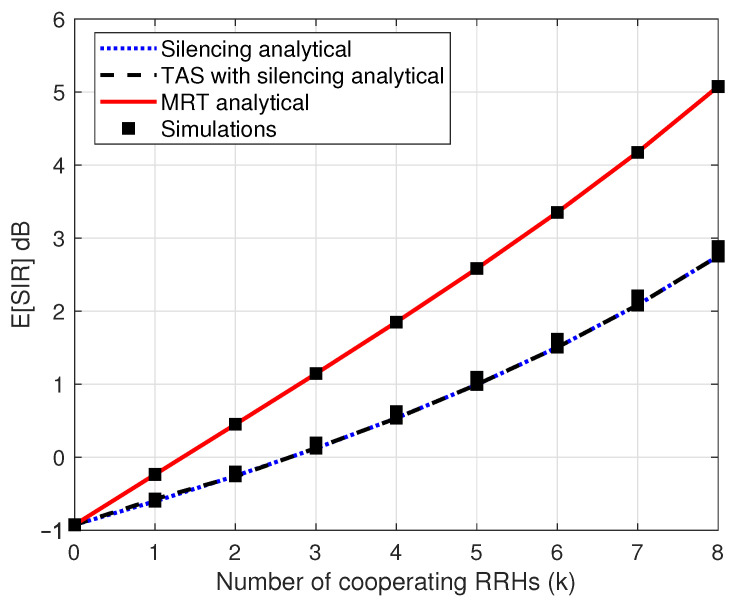
Expected value of SIR for silencing, TAS with silencing and MRT schemes as a function of number of cooperating RRHs *k*.

Figure 3 illustrates the expected value of SIR as function of number of cooperating RRHs *k* for considered transmission schemes. For all schemes, the expected SIR increases as *k* increases. While, with the same value of *k*, the expected value of SIR of MRT schemes is higher than the silencing and TAS with silencing schemes. The reason is that RRHs are jointly transmitting to the intended UE to enhance diversity gain, which increases SIR, which in turn improves the system performance. MRT helps to cope with link failure and blockages enabling multi-connectivity to serve intended UE through several cooperating links. This increment of E[SIR] with an increase in *k* improves outage performance at the tail of CDF as shown in Figure 2. Meanwhile, TAS with silencing and silencing shows very similar performance because both schemes have a single RRH transmitting to the UE, which has little diversity gain compared to MRT.

**Figure 4:**
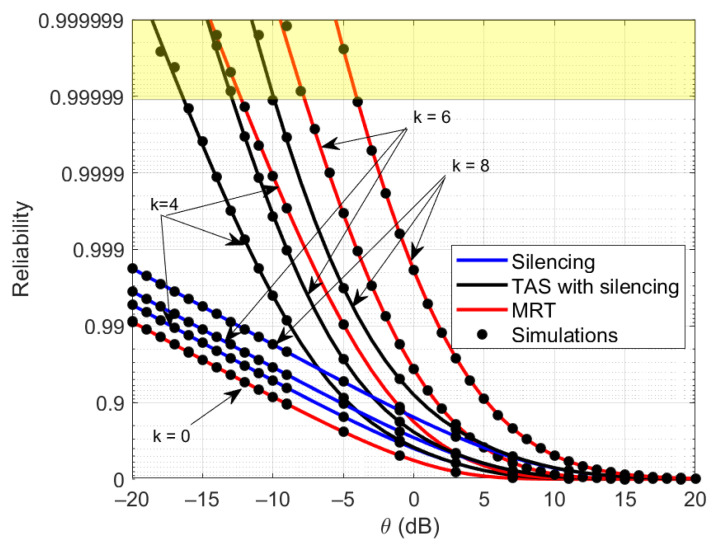
Overview of reliability analysis as a function of θ for silencing, TAS with silencing and MRT schemes with different level of *k*.

Figure 4 shows the reliability analysis as a function of θ for the different number of cooperating RRHs with silencing, TAS with silencing, and MRT schemes. The shaded region in the figure represents the ultra-reliable region of operation. In the case of k=4 RRHs in cooperation, MRT and TAS with silencing schemes achieve the ultra-reliable region. However, MRT can ensure it at higher values of θ compared to TAS with silencing. Thus, the diversity gained from MRT is superior to that of TAS with silencing schemes. This is because cooperating RRHs jointly transmit to the UE while diversifying the path loss by exploiting diverse resources. However, in silencing and TAS with silencing, this is not possible, since a typical RRH serves the UE, which has little diversity gain compared to MRT. If we consider only silencing schemes, there is a slight improvement in the reliability, with an increasing number of cooperating RRHs. However, the performance seems better with the same number of cooperating RRHs using TAS with a silencing scheme. This means silencing the same number of RRHs while using the best channel to transmit to the UE through the cooperation of RRHs can improve the reliability. For instance, when k=0, all the schemes represent full interference and have the same level of performance gain.

**Figure 5:**
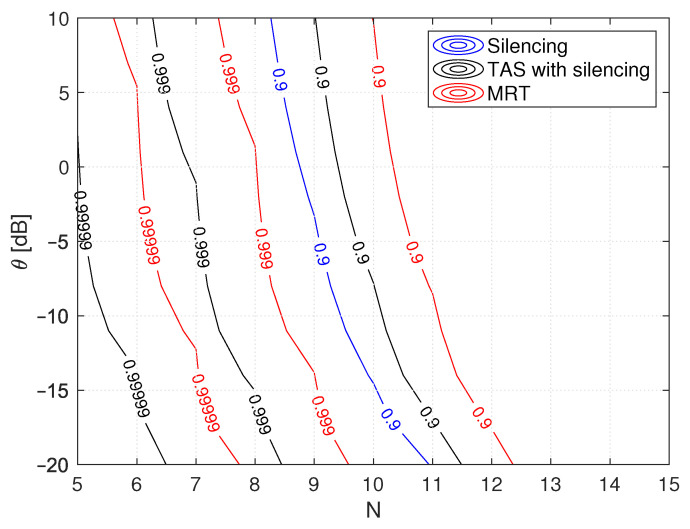
Illustration of reliability analysis for silencing, TAS with silencing and MRT schemes with respect to threshold θ as a function of *N* with fixed k=3 cooperating RRHs.

Figure 5 shows the performance analysis of silencing, TAS with silencing, and MRT schemes as a function of *N* and θ, with the fixed number of cooperating RRH k=3. As shown, the performance of MRT improves with an increase in *N* as compared to silencing and TAS with silencing because jointly transmitting from cooperating RRH or the involvement of more RRHs, provides more diversity and array gain, thus resulting in lower outage probability improving reliability. TAS with silencing outperforms silencing, but a reliability level of five nines is attainable only at a lower θ value than MRT and for smaller *N*. However, the silencing scheme attains a reliability level of one nine because of the selection of the best RRH link, which provides some additional diversity gain compared to silencing, and thus improves performance.

**Figure 6:**
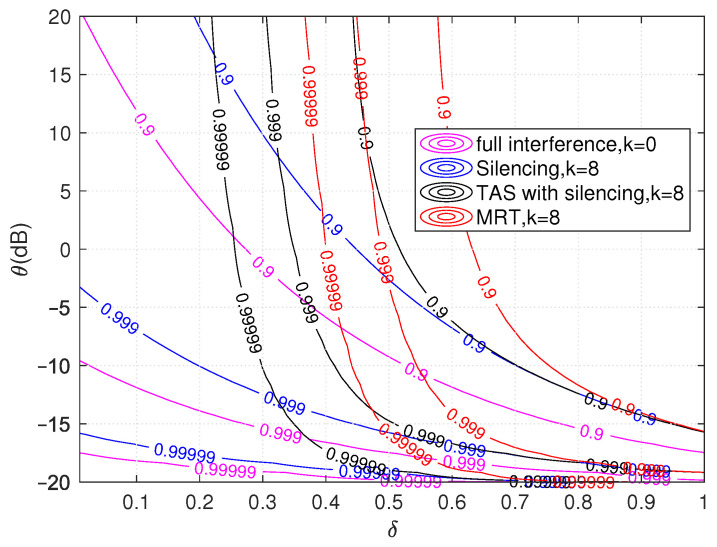
Illustration of reliability analysis for full interference, silencing, TAS with silencing and MRT schemes with respect to ratio of distances δ and threshold θ with N=10 and k=8 cooperating RRHs.

The distances from the UE to the RRHs have a significant role in this topology. We generalize the presented topology by comparing all the schemes in Figure 6 in terms of SIR threshold θ (dB) and the ratio of distances δ. The higher value of δ means the interfering and serving nodes are close to each other. For δ=0.5, the MRT schemes achieve five nines’ reliability. In contrast, TAS with silencing scheme has a reliability of double nines for a SIR threshold of 5 dB, which is not workable for silencing and full interference schemes. For example, silencing schemes attain reliability targets at lower thresholds, while MRT and TAS with silencing schemes have some higher thresholds for the same target reliability. In addition, the slope of MRT and TAS with silencing curves are verticals with higher values of δ while silencing and full interference are not. We observe that all the scheme’s performance is sensitive to distance-based interference. The improvement in reliability seems better with the MRT and TAS with silencing for the same number of cooperating RRHs compared to the silencing schemes. At the same time, the MRT has significant performance gains from joint transmission from cooperating RRHs, which shows improvement in terms of the reliability.

**Figure 7:**
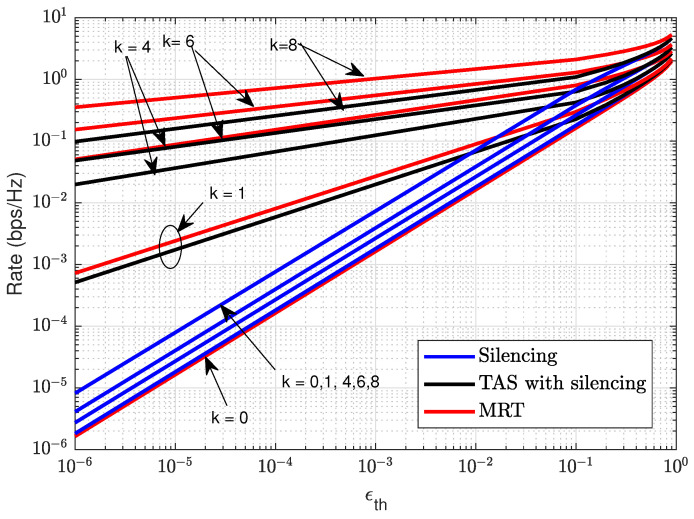
Illustration of rate control analysis with reliability constraint ($\epsilon_{th}$) for silencing, TAS with silencing and MRT schemes with N=10 and different number of cooperating RRHs *k*.

Figure 7 illustrates the target transmission rate as a function of the target reliability constraints $\epsilon_{th}$ for silencing, TAS with silencing, and MRT schemes for different levels of cooperating RRHs *k*. The target rate to attain target reliability for the silencing scheme is calculated from (11), while TAS with silencing and MRT schemes through numerical analysis from (12) and (13), respectively. We find that, as the number of cooperating RRHs (*k*) increases, there is a significant improvement over the rate in all schemes for given target reliability. However, MRT offers a higher rate for the given target reliability constraints. In comparison, TAS with silencing schemes also offer a higher rate compared to silencing schemes for the same target reliability. We see that limiting the interference with RRHs cooperation using silencing, TAS with silencing, or MRT schemes, shows a significant performance gain for target reliability constraints. As mentioned, for k=0, all the schemes operate with the same target rate. The slope of MRT curves increases with increasing cooperating RRHs *k* compared to TAS and silencing schemes because the MRT offers higher diversity gain. Even with fewer RRHs, higher data rates are achieved.

**Figure 8:**
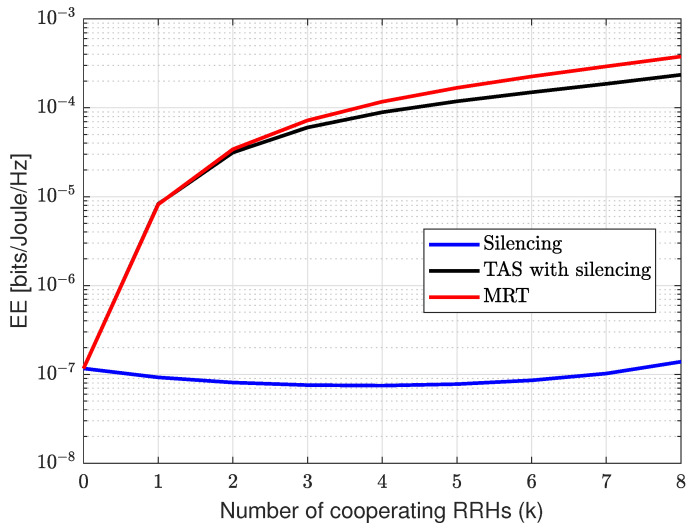
Illustration of EE for silencing, TAS with silencing and MRT with the N=10 and $\epsilon_{th}=10^{-5}$ as function of *k*.

Figure 8 presents the CRAN EE as a function of the number of cooperating RRHs for each scheme. The parameters in the power consumption model in (17) are taken from [47] where η=2.8, maximum transmit power for RRH $P_{T}=20$ Watts, active mode power for RRH $P_{i,active}^{\mathrm{RRH}}=84$ Watts, sleep mode power for RRH $P_{i,sleep}^{\mathrm{RRH}}=56$ Watts. The finding shows the improvement over the EE with the increase in cooperation for each scheme. However, from (17), power consumption increases with an increase in cooperation. As shown, the MRT scheme has EE relatively higher than TAS and silencing schemes. This is because the number of cooperating RRHs jointly transmitting to the UE increases the data rate, consequently enhancing the energy efficiency. Meanwhile, in the silencing scheme, we transmit through a single RRH, where increments over the rate is a relatively small and relatively small improvement in EE as compared with MRT and TAS with silencing. However, in the case of TAS with silencing, an increase in cooperation and selection among the best links in transmission gives a significant improvement over the rate while power consumption is the same as that of silencing schemes. Meanwhile, further increase in k>2 MRT has an improvement over EE than TAS because the data rate is higher than TAS. Note that the data rate for MRT should grow as $k\log_{2}k$, however, in this case, it grows exponentially with *k*, which is counter-intuitive. The underlying reason is that once cooperating RRHs are increased for multi-point transmission, the data rate can be realized for intended UE while guaranteeing target reliability. This is evident for small networks, thus small *k*, while for large *k*, the rate would converge to conventional cases due to the law of the large numbers. This analysis shows MRT scheme has an improvement in energy efficiency as compared with TAS and silencing schemes.

We notice that the cooperation of RRHs in interference-limited networks enhances the reliability. In this sequel, we analyze the minimum number of RRHs required to cooperate to attain target reliability by solving the argument based on CDF from (3), (6) and (9) as subjected to casse with constrained $\mathrm{Reliability}\geq 1-\epsilon_{th}$.

**Figure 9:**
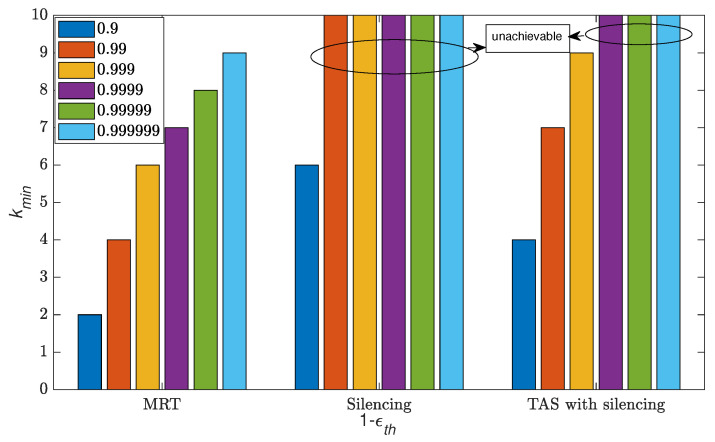
Overview of reliability levels achievable with different number of cooperating RRHs *k* for silencing, TAS with silencing and MRT schemes with the N=10.

Figure 9 shows the reliability levels achieved with the minimum number of cooperating RRHs in a CRAN service area. We used N=10 RRHs and threshold θ=0.3 dB. The analysis shows that with the MRT scheme, all reliability levels can be achieved with a different number of RRHs in comparison with silencing and TAS with silencing schemes. In the case of the reliability of five nines, which is considered as the URC operational region, only $k_{min}=8$ out of 10 are required using the MRT scheme. In contrast, such reliability is unachievable in the case of silencing and TAS with silencing schemes. In the case of TAS with silencing, the highest reliability that can be achieved with $k_{min}=9$ number of RRHs is of three nines, which can be useful in the case of serving the UE with moderate reliability. However, the only reliability level that is achieved from the silencing scheme with $k_{min}=6$ RRHs is one nine.

**Figure 10:**
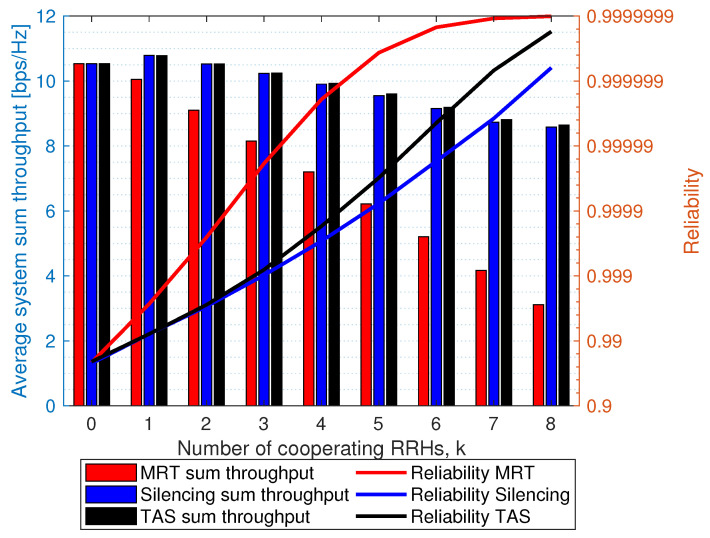
Trade-off analysis between reliability and average system sum throughput for silencing, TAS with silencing and MRT schemes as a function of *k*. We set θ=0 [dB].

We present the result of throughput reliability trade-off in Figure 10. We generate the fading channel coefficient as Rayleigh fading with unit mean. Each RRH and UE is equipped with a single antenna, and the path loss exponent is 3.5 and θ=0 [dB]. The desired distance to the UE is 20 m, while each interfering node is 40 m. Next, we choose any reference used as a stringent URLLC user and compute the corresponding reliability and average system sum throughput as output in C-RAN networks with a different number of cooperating RRHs for each scheme. As shown, there is a clear trade-off between average system sum throughput and reliability. MRT outperforms silencing and TAS with silencing schemes because cooperating RRHs simultaneously transmit to the reference user. This diversifies the path through multi-point multi-connectivity and has larger diversity gain, improving performance. Meanwhile, the average system sum throughput of the MRT scheme reduces as the number of cooperation increases. This is because cooperating RRHs cannot transmit to their own UE, consequently reducing average system sum throughput. Average system sum throughput of silencing and TAS with silencing shows a similar performance. For example, with k=1, the average system throughput rises slightly. This is because silent RRH benefits other active users in the system with reduced interference. Meanwhile, as *k* increases, we see the reduced average system throughput for fewer users being served as the RRH remains silent or cooperates to transmit. Note that the MRT average system sum throughput is small compared to TAS and silencing because other remaining active users experienced full interference, and consequently a degradation of overall average system sum throughput. For, target reliability maintaining the average system throughput, TAS seems to be a better option as compared with silencing and MRT. Note that with the k=0, i.e., full interference: all the schemes have the same average system sum throughput and reliability, which means all the RRH are actively serving their user in the network.

## 5. Conclusions

We studied the performance of the three diversity schemes: silencing, TAS with silencing, and MRT for transmission in a downlink cellular system to ensure ultra-reliable communication via CRAN. We obtained analytical closed-form expressions for the outage probability and expected value of SIR of these three schemes. The analysis showed that performance depends on the transmit rate, distance to the UE, path loss exponent, and the number of cooperating and interfering RRHs. We provided numerical results and discussion by showing outage probability and reliability analysis of the schemes when varying different system parameters. Our results showed that in the case of ultra-reliable operations, MRT schemes outperformed the TAS and silencing schemes with a minimal number of cooperating RRHs. In addition, for moderate reliability, TAS and silencing schemes are also feasible. The analysis showed that the outage probability improves by increasing the number of cooperating RRHs in an interference-limited scenario. The result showed that expected value and EE have significant improvement with increased cooperation of RRHs. In the case of EE, MRT seems beneficial compared to TAS and silencing. Meanwhile, expected values of SIR of MRT seems higher than silencing and TAS whereas silencing and TAS with silencing have very similar expected value as cooperation increases. MRT enables MC, which provides significant gain but at the cost of decreased average system sum throughput because resources are directed to the intended UE demanding ultra-reliability. Finally, as future work, we intend to extend our work focusing on the random RRHs location with Multiple-Input Multiple-Output (MIMO) scenario while considering coexisting UEs with heterogeneous quality-of-services requirements. 

## Data Availability

Not applicable.

## Appendix A. Proof of Theorem 1

In the case of silencing *k* RRHs, interference reduces to $I_{j}=\sum_{j=k+1}^{N}h_{j}d^{-\alpha }$. Then, we proceed as follows from (2)

(A1) $$\begin{array}{cc} F_{\mathrm{SIR}}S\left(\theta |q\right) & =P\left({\mathrm{SIR}}_{S}<\theta \right)=P\left(h_{0}<\theta \delta^{\alpha }q\right)\\ & =1-\exp\left(-\theta \delta^{\alpha }q\right), \end{array}$$

where $q\;\;\;=\;\;\sum_{j=k+1}^{N}h_{j}$. The last step comes from using the CDF of $h_{0}$. Next, we decondition (A1) of q using the fact that q follows a gamma distribution [49] with PDF $f_{Q}\left(q\right)=\frac{q^{\left(N-k-1\right)}e^{-q}}{(N-1)!}$. Thus, we proceed to calculate the CDF as

(A2) $$\begin{array}{cc} F_{{\mathrm{SIR}}_{S}}\left(\theta \right) & =\int_{0}^{\infty }\;F_{{\mathrm{SIR}}_{S}}\left(\theta |q\right)f_{Q}\left(q\right)\mathrm{dq}\\ & \overset{\left(a\right)}{=}\;1\;-\;\int_{\;0\;}^{\;\infty \;}\;\exp\left(\;-q\delta^{\alpha }\theta \;\right)\;\frac{q^{N-k-1}}{(\;N-k-1\;)!}\mathrm{dq}\\ & \overset{\left(b\right)}{=}\;1\;-{\left(\frac{1}{\;1+\delta^{\alpha }\theta }\;\right)}^{N-k}\frac{1}{(\;N\;-\;1\;)!}\int_{0}^{\infty }e^{\;-x\;}x^{\;N-1\;}dx\\ \; & \overset{\left(c\right)}{=}1-{\left(1+\delta^{\alpha }\theta \right)}^{k-N}, \end{array}$$

where (*a*) comes from substituting the respective CDFs. Also, we substitute the exponent of the exponential in (*a*) with *x* follows for integral limit as q→∞ then x→∞. Similarly, when q→0 then x→0 where $\mathrm{dq}=\frac{dx}{1+\delta^{\alpha }\theta }$ and the following equation (*a*) reduced to form (*b*). The integral in (*b*) is the definition of gamma function and reduces Γ(N)=(N−1)! since $N\in N^{*}$, which simplifies into (*c*), that is the closed form solution for silencing scheme concluding the proof.

## Appendix B. Proof of Theorem 2

In the case of TAS with silencing scheme, we can rewrite (5) as

(A3) $$\begin{array}{c} F_{{\mathrm{SIR}}_{\mathrm{TAS}}}\left(\theta \right)=P\left(\frac{\Omega }{\sum_{j=k+1}^{N}h_{j}d^{-\alpha }}<\theta \right), \end{array}$$

where,

(A4) $$\begin{array}{c} \Omega =\max(h_{0}d_{0}^{-\alpha },\left(h_{1}......h_{k}\right)d^{-\alpha }). \end{array}$$

Note that in (A4), *k* is the number of RRHs in cooperation, and we take the maximum between typical and interfering RRHs in cooperation. Now from (A3) and (A4) we have that

(A5) $$\begin{array}{c} F_{{\mathrm{SIR}}_{\mathrm{TAS}}}\left(\theta \right)=P\left(\frac{\max(h_{0}d_{0}^{-\alpha },\left(h_{1}\ldots \ldots h_{k}\right)d^{-\alpha }}{Zd^{-\alpha }}<\theta \right). \end{array}$$

Let $Z=\sum_{j=k+1}^{N}h_{j}$ be a gamma distributed RV such that Z∼Γ(k,N). Then we can rewrite (A5) conditioned on Z as

(A6) $$\begin{array}{cc} F_{{\mathrm{SIR}}_{\mathrm{TAS}}}|{}_{Z}\left(\theta \right) & =P\left(\max\left(h_{0}d_{0}^{-\alpha },\left(h_{1},\cdots ,h_{k}\right)\right)d^{-\alpha }<\theta Zd^{-\alpha }|Z\right), \end{array}$$

From the definition of CDF and higher order statistics [50] we rewrite (A6) as

(A7) $$\begin{array}{c} F_{{\mathrm{SIR}}_{\mathrm{TAS}}}|{}_{Z}\left(\theta \right)=(1-\exp\left(-\theta Z\delta^{\alpha }\right){\left(1-e^{-\theta Z}\right)}^{k}. \end{array}$$

Then, de-conditioning (A7), we have

(A8) $$\begin{array}{cc} F_{{\mathrm{SIR}}_{\mathrm{TAS}}}\left(\theta \right) & =\int_{0}^{\infty }\left(1-\exp\left(-\theta Z\delta^{\alpha }\right)\right)\underset{\left(a\right)}{\underset{︸}{{\left(1-e^{-\theta Z}\right)}^{k}}}f_{Z}\left(z\right)\mathrm{dZ}. \end{array}$$

By using binomial Theorem, (1+x)n=∑k=0nnkxk to expand term (a) in (A8) and substituting the PDF of Z, which is gamma distributed as $f_{Z}\left(z\right)=\frac{Z^{\left(N-k-1\right)}e^{-Z}}{(N-1)!}$ we have that,

(A9) FSIRTAS(θ)=∑t=0kkt∫0∞1−exp(−θZδα)(−1)t×(e−θZ)tZN−k−1e−Z(N−k−1)!dZ.

Thus, simplifying and substituting x=Z(1+θt) and $y=Z(1+\theta t+\theta \delta^{\alpha })$ and setting limits as x,y→0 when Z→0 and x,y→∞ when Z→∞ respectively into (A9), we have

(A10) FSIRTAS(θ)=∑t=0kkt1(N−k−1)!(−1)t((1+θt)k−N×∫0∞e−xxN−k−1dx−(1+θt+θδα)k−N×∫0∞e−yyN−k−1dy).

Simplifying the gamma function in (A10), we can achieve our closed form solution for TAS with silencing scheme concluding the proof as

(A11) FSIRTAS(θ)=∑t=0kkt(−1)t(1+θt)k−N−1+θt+θδαk−N.

## Appendix C. Proof of Theorem 3

In the case of MRT schemes, we proceed from (8) as

(A12) $$\begin{array}{cc} F_{{\mathrm{SIR}}_{\mathrm{MRT}}}\left(\theta |h_{c},h_{j}\right) & =P\left({\mathrm{SIR}}_{\mathrm{MRT}}<\theta |h_{c},h_{j}\right)=P\left(h_{0}<\delta^{\alpha }\left(\sum_{j=k+1}^{N}\theta h_{j}-\sum_{c=1}^{k}h_{c}\right)|h_{c},h_{j}\right)\\ \; & =1-\exp\left(-\delta^{\alpha }\left(\theta \sum_{j=k+1}^{N}h_{j}-\sum_{c=1}^{k}h_{c}\right)\right) \end{array}$$

where the last step comes from using the CDF of $h_{0}$. Next, we de-condition (A12) of $h_{c}$ and $h_{j}$ using the fact that $p=\sum_{c=1}^{k}h_{c}∼\Gamma \left(k,\frac{1}{k}\right)$ and $q\;=\;\sum_{j=k+1}^{N}h_{j}∼\Gamma \left(N-k,\frac{1}{N-k}\right)$. Thus, we proceed to calculate CDF as

(A13) $$\begin{array}{c} F_{{\mathrm{SIR}}_{\mathrm{MRT}}}\left(\theta \right)=\int_{0}^{\infty }\int_{\frac{p}{2^{r}-1}}^{\infty }\;(1-\exp\;\left(-\delta^{\alpha }\left(\theta p-q\right)\;)\right)f_{q}\left(q\right)f_{p}\left(p\right)\mathrm{dq}\mathrm{dp}. \end{array}$$

The integral in (A13) comes after applying the CDF of $h_{0}$ and de-conditioning on p and q. From definition of hyper-geometric function [37], we solve (A13) and obtain (9). Note that (9) is our final closed-form solution for δ<1, while δ>1 is neglected since the UE connects to the closest RRH thereby concluding the proof.
